# Mathematical modelling of the waning of anti-RBD IgG SARS-CoV-2 antibody titers after a two-dose BNT162b2 mRNA vaccination

**DOI:** 10.3389/fimmu.2023.1097747

**Published:** 2023-01-26

**Authors:** Francisco J. Cimas, Javier Torres, Jesús Ontañón, Carlos de Cabo, Julia Lozano, María Ángeles Requena, Joaquín Blas, José Luis Rodríguez-García, Antonio Mas, Javier Solera

**Affiliations:** ^1^ Mecenazgo COVID-19, Faculty of Medicine/Centro Regional de Investigaciones Biomedicas (CRIB), University of Castilla-La Mancha at Albacete, Albacete, Spain; ^2^ Clinical Analysis Department, Albacete General Hospital, Ciudad Real, Spain; ^3^ Immunology Unit, Albacete General Hospital, Albacete, Spain; ^4^ Research Department, Albacete General Hospital, Albacete, Spain; ^5^ Microbiology Department, Albacete General Hospital, Albacete, Spain; ^6^ Internal Medicine Department, Albacete General Hospital, Albacete, Spain; ^7^ Molecular Virology Laboratory, Department of Medical Sciences, Faculty of Medicine/Centro Regional de Investigaciones Biomedicas (CRIB), University of Castilla - La Mancha at Albacete, Ciudad Real, Castilla-La Mancha, Spain

**Keywords:** COVID-19, SARS-CoV-2, vaccines, IgG, immunity, hybrid immunity

## Abstract

**Background:**

After exposure to SARS-CoV-2 and/or vaccination there is an increase in serum antibody titers followed by a non-linear waning. Our aim was to find out if this waning of antibody titers would fit to a mathematical model.

**Methods:**

We analyzed anti-RBD (receptor binding domain) IgG antibody titers and the breakthrough infections over a ten-month period following the second dose of the mRNA BNT162b2 (Pfizer-BioNtech.) vaccine, in a cohort of 54 health-care workers (HCWs) who were either never infected with SARS-CoV-2 (naïve, nHCW group, n=27) or previously infected with the virus (experienced, eHCW group, n=27). Two mathematical models, exponential and power law, were used to quantify antibody waning kinetics, and we compared the relative quality of the goodness of fit to the data between both models was compared using the Akaik Information Criterion.

**Results:**

We found that the waning slopes were significantly more pronounced for the naïve when compared to the experienced HCWs in exponential (p-value: 1.801E-9) and power law (p-value: 9.399E-13) models. The waning of anti-RBD IgG antibody levels fitted significantly to both exponential (average-R2: 0.957 for nHCW and 0.954 for eHCW) and power law (average-R2: 0.991 for nHCW and 0.988 for eHCW) models, with a better fit to the power law model. In the nHCW group, titers would descend below an arbitrary 1000-units threshold at a median of 210.6 days (IQ range: 74.2). For the eHCW group, the same risk threshold would be reached at 440.0 days (IQ range: 135.2) post-vaccination.

**Conclusion:**

Two parsimonious models can explain the anti-RBD IgG antibody titer waning after vaccination. Regardless of the model used, eHCWs have lower waning slopes and longer persistence of antibody titers than nHCWs. Consequently, personalized vaccination booster schedules should be implemented according to the individual persistence of antibody levels.

## 1 Introduction

The COVID-19 pandemic has represented a huge challenge for societies and health systems all over the world. While there are more and more people with an immune shield, either due to recovery from primary SARS-CoV-2 infection, vaccination or both (1), the virus has continued to evolve with the emergence of new genetically distinct variants that imply a higher transmission rate and a decrease in the immune protection against re-infection (2–4)

Vaccines against SARS-CoV-2 have demonstrated a high degree of protection against COVID-19 over time, at least in terms of severe disease and mortality (5, 6). However, immunity to SARS-CoV-2 declines in a nonlinear fashion (7, 8) and makes it difficult to estimate risk and make decisions about when to schedule booster vaccines (9). In fact, it has already been described that antibody (Ab) titers correlate with immune protection, and they have been used to predict protection against infection (10–14). This decrease in protection is different in vaccinated subjects without previous infection (naïve) versus previously-infected (experienced) vaccinated subjects due to hybrid immunity (1, 15, 16). In this work, we monitored anti-RBD IgG Ab titers and registered the breakthrough infections from February 2021 to December 2021, just before the third vaccination dose, in a cohort of health care Workers (HCWs) vaccinated with the mRNA BNT162b2 (Pfizer BioNtech.) vaccine in January 2021. We proposed that it was possible to model waning in anti-RBD Ab titer levels over time through simple mathematical models. We used exponential and power law models, which accurately describe non-linear waning of antibody titers for both natural infection and vaccination (11, 17–20). Our aim was to develop a simple way to describe evolution of the antibody titer over time. This would allow us to predict a personalized optimal moment to administer of the booster vaccine dose as well as estimate risk of infection and its severity.

## 2 Materials and methods

### 2.1 Study design and overview

We conducted an observational prospective longitudinal study based on a previously reported HCWs cohort from the Albacete General Hospital (CHUA, Spain) (21). This study was officially approved by the Comité de Ética de la Investigación con medicamentos (CEIm) de la Gerencia de Atención Integrada de Albacete (Internal code: 2021-12 EOm). Written informed consent was obtained from all study participants. These HCWs were vaccinated in January 2021 with the BNT162b2 mRNA vaccine. Briefly, 63 HCWs from the original CHUA cohort volunteered to measure their antibody levels at several time-points during three periods. The first period included one measurement before the onset of vaccination. During the second period, measurements were taken at 7, 14 and 21 days following each of the two vaccine doses, while during the third phase, monthly measurements were performed until administration of the third vaccine dose (December 2021). Of the 63 original HCWs, we included 54 subjects who complied with the follow-up schedule; at least 4 decreasing consecutive measurements after completing the vaccination schedule. Subjects were classified into two groups: naïve health-care workers (nHCWs), which included participants without clinical or laboratory data suggestive of infection with the SARS-CoV-2 virus prior to vaccination, and experienced health-care workers (eHCWs), consisting of those with previous SARS-CoV-2 infection. The kinetics of antibody decay was evaluated for each subject, considering the point of maximum antibody level (between 7 and 45 days after the second vaccination dose) and all the determinations made during the following 10 months. We monitored the eventual appearance of breakthrough symptomatic or asymptomatic infections and their symptomatology. Breakthrough infections were defined as the detection of SARS-CoV-2 by PCR 14 or more days after receiving the second dose.

### 2.2 Biochemical analysis

Total IgG antibody levels against the S1 subunit of the SARS-CoV-2 virus spike protein that binds to the receptor binding domain (RBD) were measured using the SARS-CoV-2 IgG II Quant immunoassay in the ARCHITECT i-System (Abbott, Abbott Park, IL, USA). The analytical measurement range is from 21 to 80,000 AU/mL and we used the manufacturer’s recommended cutoff point of 50.0 AU/mL to determine positivity. To convert AU/mL into international standard WHO units (BAU/mL) the conversion factor is 1/7 (22). To assess re-infection detection, PCR was performed in samples of nasopharyngeal exudates that were collected in tubes with 3 mL of universal transport medium (UTM) without inactivation and routinely sent to our laboratory for diagnosis of SARS-CoV-2. Samples were extracted with MagMaxTM Viral/Pathogen Nucleic Acid Isolation Kit (ThermoFisher) reagents using the KingFisher extractor (ThermoFisher) following manufacturer instructions. For the qualitative detection of SARS-CoV-2 nucleic acid, the commercial TaqPathTM COVID-19 CE-IVD Kit was used together with the ThermoFisherQuantStudio 5 (QS5) thermal cycler.

### 2.3 Statistical analysis

Quantitative demographic variables were expressed as mean and range or mean and standard deviation (SD) and with a confidence interval of 95% (CI). Qualitative variables were expressed as number and percentage. Total anti-SARS-CoV-2 spike RBD region IgG antibody levels were reported using geometric mean concentrations (GMC).

Normality of the distributions was tested using Lilliefors test and variances between populations using the F-test. The two-tailed U-Mann-Whitney non-parametric method was used to compare different means between the nHCW and eHCW groups. Within-group differences in total IgG levels obtained at the different time points were assessed using the two-tailed Wilcoxon Sign test. Chi-square and Fisher’s test were used to compare categorical data. All the confidence intervals, as well as the statistical tests, were calculated with a significance level of 95%.

Two mathematical models were used to quantify antibody waning kinetics in each patient. Firstly, the exponential model y = *α* ·e^(*β* ·x) was employed, for each patient, where y is the SARS-CoV-2 RBD IgG antibody concentration, *α* the exponential transformation of the extrapolated SARS-CoV-2 IgG concentration of the HCW at day 0, *β* the slope of the model, and x the time after vaccination in days. The parameters of the model were estimated by fitting a linear model to the Naperian logarithm of the SARS-CoV-2 RBD IgG concentration versus the time after vaccination expressed in days, using the Ordinary Least Squares method. The exponential curve was subsequently obtained by reversing the logarithmic transformation.


$$\left(\text{RBD IgG}-\text{SARS}-\text{CoV}-2\right)=\;\alpha \cdot e^{\beta \cdot \left(time\;in\;days\right)}$$

 Ln (RBD IgG−SARS−CoV−2)= Ln(α)+ β·(time in days)

Equation 1: Exponential and linear representations of concentration of IgG versus time.

Secondly, a power law model was also used, represented by the curve y = *α* · x^ *β* employing the same variable definitions as before. The adjustment was performed by Ordinary Least Squares regression of the Napierian logarithm of the concentration of SARS-CoV-2 IgG concentration versus the Napierian logarithm of time. Exponential transformation was performed to obtain the curve from the linear adjustment:


Ln(RBD IgG−SARS−CoV−2)= α+ β ·Ln(time in days)

$$\left(\text{RBD IgG SARS}-\text{CoV}-2\right)=e^{\alpha }\;{\left(time\;in\;days\right)}^{\beta }$$

Equation 2: Power law and linear representations of the concentration of IgG versus time.

For both models, the relative quality of the goodness of fit to the data between both models was compared using the Akaike Information Criterion (AIC) (23). AIC was calculated for each patient and model. The difference in the AIC value was calculated as the exponential model’s AIC minus the power law model’s AIC. Positive delta values were interpreted as a better exponential fit and negative values as a better power law fit.

An average waning curve was constructed for each mathematical model and sub-cohort by calculating the mean values of the individual fitted curves, obtaining an average curve of the individual ones. Calculations were carried out with the statistical software R, version 4.0.2 and data visualization figures were drawn with the ggplot2 package.

## 3 Results

### 3.1 Characteristics of the study population

54 HCWs were included in the study, of which 27 were nHCWs and 27 eHCWs regarding SARS-CoV-2 infections previous to vaccination. The epidemiological characteristics of participants are described in **Table 1**. No significant differences were found in terms of age, sex, number of Ab-titer determinations (timepoints) and follow-up duration between the two groups.

**Table 1:** Demographic characteristics of the participants in the study: * U-Mann-Whitney test; ** Chi Square test.

	nHCW	eHCW	p-value
Number	27	27	
Age in years. Mean ± SD (Range)	42.0 ± 14.91 (25–62)	47.96 ± 12.78 (25–67)	0.12 *
Number of females (%)	19 (70%)	19 (70%)	1.00 **
Follow-up days after the second dose of the vaccine. Mean ± SD (Range)	293.52 ± 3.50 (21–27)	293.07 ± 10.82 (21–28)	0.84 *
Number of determinations per subject. Mean ± SD (Range)	6.85 ± 2.35 (4–12)	6.33 ± 1.33 (4–10)	0.32 *

### 3.2 A differential decrease in post-vaccine Ab titers was observed between nHCWs and eHCWs

The geometric mean of the maximal post-vaccine values for the eHCW group was two times higher than the nHCW group (46.682 AU/mL Vs 23.623 AU/mL, p-value< 0.001).

**Figure 1:**
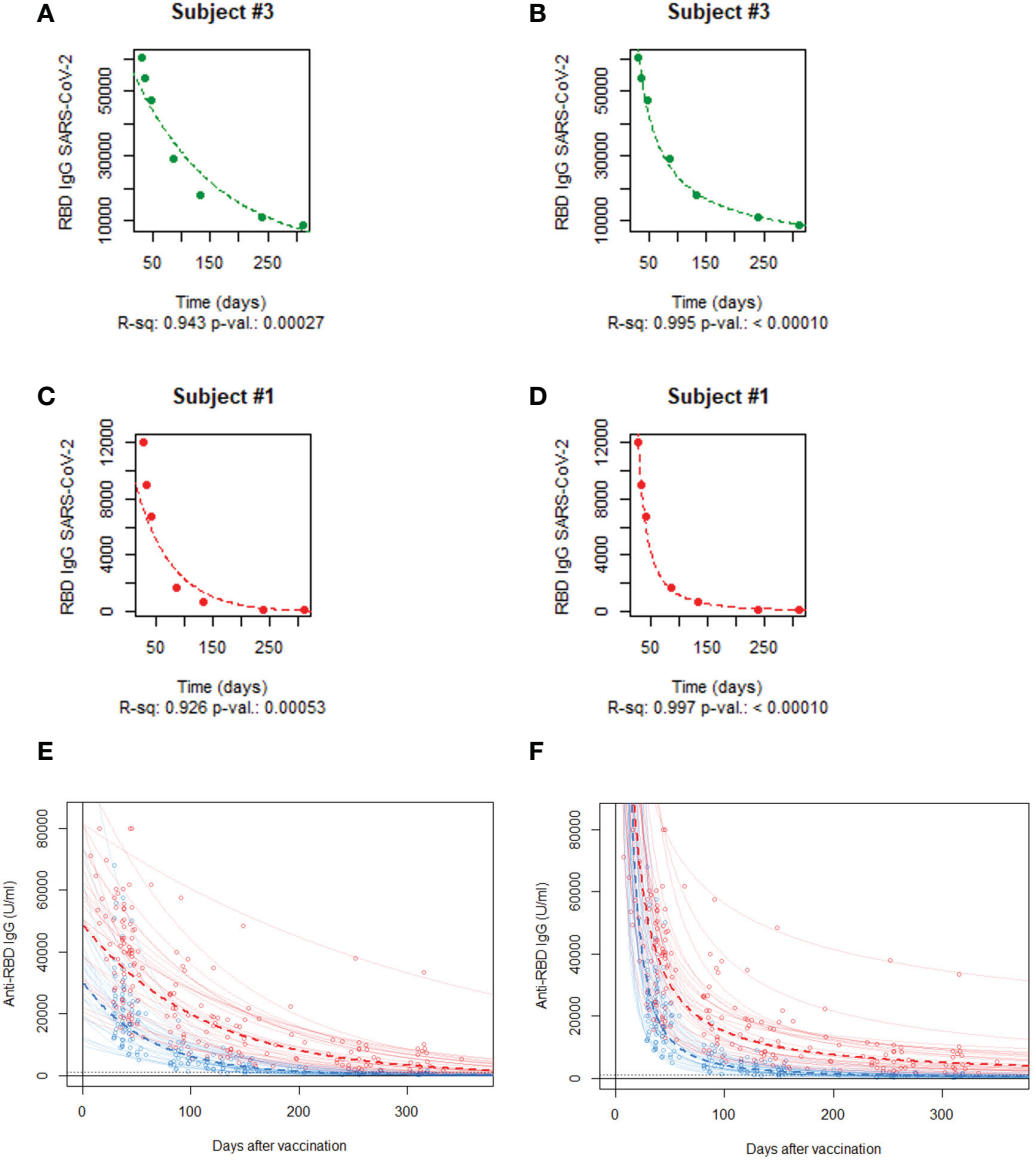
Time-course of anti-RBD IgG antibody titers. Representative individual anti-RBD IgG antibody titer curves of a naïve HCW for exponential **(A)** and power law **(B)** models. Representative individual anti-RBD IgG antibody titer curves of an experienced HCW for exponential **(C)** and power law **(D)** models. Mean curves for exponential **(E)** and power law **(F)** models. Individual timepoints for each HCW are represented as open circles (red, eHCWs and blue, nHCWs). Each continuous line represents an ideal antibody waning curve for individual HCWs; dotted lines represent the ideal mean curves. The horizontal dotted line indicates 1.000 AU/mL.

We plotted the individual Ab waning curves from the measured Ab titers for each HCW using both exponential and power law models (**Figures 1A–D**; **Supplementary 1**). We observed that after reaching maximum post-vaccine values, the anti-RBD Ab titer levels decreased over the following 10 months in both the naïve and eHCW groups (**Figures 1E, F**). The waning of the curves for the nHCWs were more pronounced when compared with the eHCWs in both the exponential and power law models.

Firstly, for each HCW individual data were fitted to an exponential curve, applying a linear regression method. Within the nHCW group, the decay rate *β* had a median value of -0.015736 (Interquartile range: -0.019122 to -0.012970) and a mean value of -0.016137 (SD: 0.00412; 95% CI: -0.017692 to -0.014582). The R2 values for all the adjustments were between 0.899 and 0.996, with an average of 0.957 which demonstrates a good fit of the curve for every individual to their experimental data. All the p-values for the adjustments were significant (p-value range: 9.18E-7 to 5.0E-3) (**Figure 1A**). For the eHCW group, the median *β* parameter was -0.008496 (Interquartile range: -0.009983 to -0.007256) and the mean value -0,009054 (SD: 0,00299; 95% CI: -0.01018 to -0.0079). Their calculated curves showed a good fit to the experimental data, similar to the one found for the nHCW group (**Figure 1A**). The R2 value for the adjustments was between 0.907 and 0.998, with an average value of 0.954. All the corresponding p-values were significant (p-value range: 1.406E-7 to 0.844E-3).

Secondly, in the case of the power law fit, for the nHCW group, the parameter *β*, corresponding to the linear regression slope representing the Ln of SARS-CoV-2 IgG concentration against the Ln of time had a median value of -1.598 (Interquartile range: -1.803 to -1.403) and a mean value of -1.623 (SD: 0.240; 95% CI: -1.713 to -1.523). A good fit of the curve to the experimental data was found for each individual (**Figure 1B**). The R2 values for all the adjustments were between 0.970 and 0.999, with an average of 0.991. All the p-values for the adjustments were significant (p-value range: 1.31E-9 and 2.27E-3). Regarding the eHCW group, analysis showed a median parameter *β* of -0.977 (Interquartile range: from -1.161 to -0.877) and a mean value of -1.005 (SD: 0.247; 95% CI: -1.098 to -0.912). The calculated curves demonstrated a similarly good fit to the experimental data of the nHCWs group (**Figure 1B**). The R2 value for the adjustments was found to be between 0.931 and 0.9997, with an average value of 0.988. All the corresponding p-values were significant (p-value range: 4.91E-12 and 7.91E-3).

**Figure 2:**
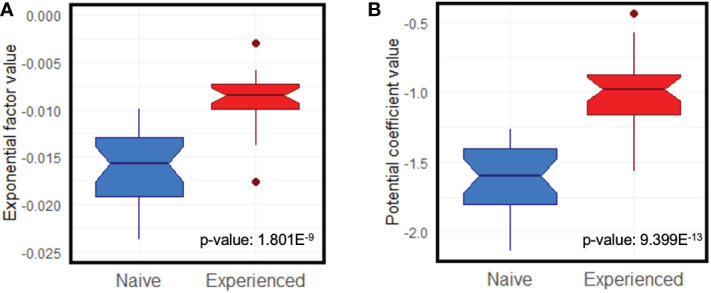
Notched box and whisker comparison of the ideal curves slope means for exponential **(A)** and power law **(B)** models. Box width is proportional to the number of observations in each group (red, eHCWs and blue, nHCWs).

The slopes of the average curves for the nHCWs were more pronounced when compared with the eHCWs in the exponential (1.801E-9) and power law models (p-value: 9.399E-13) (**Figure 2**). Comparison of both models was calculated for each individual HCW using the value of the AIC difference for the exponential minus power law model (deltaAIC) (**Supplementary 3**). This difference was positive for 46/54 HCW (85.2%), denoting a better fit for the power law model.

### 3.3 Characterization of breakthrough infections in the studied cohort

Of the 54 HCWs included in this study, only 4 (7.4%) had breakthrough infections (**Table 2**). Although 3 of them (15%) belonged to the nHCW group and just 1 (5%) to the eHCW group, no statistically significant differences were found (p-value = 0.6104). Two of nHCWs were infected in July 2021, when the predominant SARS-CoV-2 variant was delta and the other two infections occurred in December 2021 when the predominant variant was Omicron. We estimated the anti-RBD antibody levels at the time of infection for all four HCWs. For the nHCW the estimated value was< 1000 AU/mL, with a mean value of 504.2 AU/mL; maximum and minimum values were 932.6 AU/mL and 15.0 AU/mL, respectively. For the eHCW, the anti-RBD antibody levels at the time of infection were< 4000 AU/mL (**Table 2**).

**Table 2:** Parameters of interest of the breakthrough infection cases detected during our study.

Subject	Group	Sex	Previous diagnosis	2nd dose date (month/year)	Break. inf. date	Titer at break infect. (Expo./Pow. law)	R2 (Expo./Pow. law)
54	N	F	X	02/2021	07/2021	563.9/1265.9	0.9715/0.9866
55	N	M	X	02/2021	07/2021	932.6/2276.6	0.9743/0.9764
48	N	F	X	02/2021	11/2021	16.0/527.8	0.9734/0.9956
26	P	F	03/2020	02/2021	12/2021	2960.5/4029.5	0.955/0.9769

Parameters include group, sex, existence of previous diagnosis, 2nd dose date, breakthrough infection date (in month/year format), anti-RBD IgG titer at breakthrough infection time and adjusted R2 (calculated by exponential model/calculated by power law model).

### 3.4 Usefulness of the model to predict Abs levels and use of the data obtained in decision making

For the nHCW group, the post-vaccination half-life of the RBD-Ab titers estimated by the exponential model was 45.84 days on average (SD: 12.09, 95% CI: 41.28-50.41) for the nHCW group and, whereas for the eHCW group, the half-life was 85.67 days (SD: 34.95; 95% CI: 72.48 to 98.85 days). This result for eHCWs is 1.87-fold higher than for the nHCWs group.

We established an arbitrary risk threshold of 1000 AU/mL anti-RBD Ab titer for symptomatic infection based on the upper titer threshold for volunteers who suffered infection prior to the Omicron outbreak. In the nHCW group, titters were predicted to descend to this 1000-units threshold at 221.1 days post-vaccination, on average (SD: 53.1 days). The median for this group was 210.6 days (Interquartile range: 74.2). However, for the eHCW group, the risk threshold would be reached at 483.3 days post-vaccination, on average (SD: 229.1 days). For this group, the median was located at 440.0 days (Interquartile range: 135.2). The difference in the predicted mean time to descend to the risk threshold between nHCWs and eHCWs was statistically significant (p-value = 2.14E-12, Mann-Whitney U-test). Interestingly, in one individual from the eHCW group, a value below the 1000 AU/mL risk threshold would be reached as late as 1.468,9 days. Predictions made with the power law model develop asymptotic behavior after long periods and are reflected on **Supplementary Table 2**.

According to the extrapolations made, when 95% of the nHCWs group would have already fallen below this limit, only 5 (18.5%) of the eHCWs would have done so, while the remaining 22 (81.5%) would still be above the threshold. Similarly, when 50% of eHCW patients would be below the threshold, all of the nHCW patients would already dropped be below the limit.

## 4 Discussion

In this study, we modelled the observed waning of anti-RBD IgG Ab levels over a 10-month period in a cohort of HCWs following a second dose of the BNT162b2 vaccine. Two different mathematical approximations, the exponential and power law models, were employed. Both models fitted well, with strong correlation coefficients and statistical significance (**Supplementary Figure 1**). Thanks to the goodness of fit of the curves to both power law and exponential models, we would be able to predict individual Ab titers following the second dose of the mRNA BNT162b2 vaccine with only two Ab determination timepoints.

Interestingly, the power law model demonstrates a better fit for the earlier determinations, consistent with the quick waning in Ab titers during the first weeks following peak levels. This observation is consistent with previous reports describing a rapid decline in SARS-CoV-2 IgG Ab titer during the first four months after antigen contact, followed by a gentler waning over the subsequent 7 months (11, 12, 24). At this point, Ab levels correlate with the presence of antigen-specific plasma cells found in bone marrow (25–27). The power law model also presents a better fit than the exponential model according to the AIC criterion. A negative delta value was found for 85% of subjects (**Supplementary Table 1**). The exponential and power law models have been previously compared to describe anti-RBD IgG titer waning in a smaller, naïve cohort (11). That same study found a more robust fitting of the power law model to the anti-RBD Ab waning curve, similarly to our results. However, they found a better fit for the exponential model when analysing other determinations of neutralizing Ab.

The nHCW and eHCW sub-cohorts have a statistically significant difference in slopes, showing a pronounced decline for nHCWs in both models. Multiple studies have shown that vaccinated subjects with previous infection have an early, stronger and longer lasting response to vaccines than naïve ones, as well as lower risk of infection (1, 6, 12, 15, 16, 28, 29); this is attributed to hybrid immunity. Therefore, our results agree with the hypothesized lower risk of infection for hybrid immunity bearers. Compared to previous works, our study provides a detailed Ab titer evolution throughout more timepoints and also demonstrates significant differences between the nHCW and eHCW sub-cohorts slopes.

During follow-up, we detected breakthrough infections in 4 HCWs; all of them were mild and did not require hospitalization. Three of them corresponded to nHCWs and only 1 to eHCWs. The number of infections was higher for nHCWs, as has been widely described (1, 15, 16). However, no statistical significance was found due to the reduced number of relapse cases given the small size of the cohort. With that in mind, those HCWs who experienced relapses had lower Ab levels at the time of infection. Conversely, the average Ab levels of those who did not relapse were higher than for those who did. The level of Abs against SARS-CoV-2 can be a marker of protection or risk (a correlate of protection) to determine individual risk of infection and the optimal moment for a booster vaccine; however, cellular immunity should also be taken into account (30). In this regard, testing our model in larger cohorts with more breakthrough infections would allow us to estimate risk of infection thresholds and correlates of protection more accurately.

Notwithstanding the above-mentioned goodness of fit of the models, we observed a high individual variability among the curves, that was more pronounced in nHCWs (**Figure 1**) It is remarkable that, within the two well-differentiated groups in terms of presence or absence of hybrid immunity, there was such a high level of heterogeneity in the anti-RBD Ab titer curves. This variability ranged from vulnerable subjects with sustained low level antibody titers to others who were able to maintain high antibody titers over time, conferring protection against infection. We found one eHCW in particular with such high, sustained Ab titers, that a booster vaccination would be unnecessary for a long time. These two types of responders to vaccination (low and high), have been well characterized by Nakamura et al. (24) in a larger cohort. The variability in the anti-RBD IgG antibody response supports the use of individual curves versus mean curves when attempting to predict Ab titers over time.

In terms of limitations, our study was conducted in a small, homogeneous cohort of HCWs, who were mostly middle-aged and healthy subjects and therefore may not represent the general population. Moreover, due to the period in which the study was performed, no new SARS-CoV-2 variants nor other vaccines or doses patterns were considered. Our model needs rigorous evaluation using data from different cohorts. Besides, antibody level estimations with our model for dates outside our period of study should be taken with caution. This model, like many other predictive models, is based on multiple regression techniques. In a recent study, this approach was combined with machine learning (19), which we did not use in our work. Nonetheless, in our study, we did describe detailed Ab individual waning curves comparing eHCWs with nHCWs at several timepoints and for a similarly long follow-up period.

In conclusion, we described both exponential and power law models as parsimonious models that allow determination of the anti-RBD IgG Ab titer, building a personalized waning curve with only two antibody titer determinations. Therefore, we can estimate the moment when antibody titers drop below a certain threshold. Nevertheless, it should be noted that predictions would be most reliable within the 10-month observation period used in this work. Regardless of the model used, eHCWs have a lower waning slope and longer persistence of antibody titers than nHCWs. Consequently, different vaccination booster schedules should be implemented according to individual persistence of antibody levels. Our modelization could also be used under different conditions which may alter conferred protection, such as new vaccines or viral variants. Our approach provides a tool for personalized predictions of Ab levels, and, thus, the rationalization of booster dose administration of anti-SARS-COV-2 vaccines, applying them only when necessary and avoiding potential side-effects.

## Data availability statement

The raw data supporting the conclusions of this article will be made available by the authors, without undue reservation.

## Ethics statement

The studies involving human participants were reviewed and approved by Comité de Ética de la Investigación con medicamentos de la Gerencia de Atención Integrada de Albacete. The patients/participants provided their written informed consent to participate in this study.

## Author contributions

Conceptualization: JS, JO, FC, CC, AM JR-G. Formal analysis: JT, JI, JL, MR, JB, FC. Investigation: JT, JO, JL, MR, JB, FC. Verification of the underlying data: JT, JO, JL, JB, FC. Writing- original draft preparation: JO, FC, CC, JR-G, JS. Writing-review and editing: JO, FC, CC, JR-G, JS. Supervision: FC, AM, JO, JS, CC. All authors had full access to all of the data in the study and accept responsibility for submission of the work for publication. All authors contributed to the article and approved the submitted version.

## Supplementary material

Supplementary 1 Time-course of anti-RBD IgG antibody titers for each individual HCW. **(A)** Individual time-courses of naïve subjects adjusted by exponential model. **(B)** Individual time-courses of experienced subjects adjusted by exponential model. **(C)** Individual time-courses of naïve subjects adjusted by power law model. **(D)** Individual time-courses of experienced subjects adjusted by power law model.

Supplementary 2 Linear transformation of individual curves for both nHCW (in blue) and eHCW (in red) groups for the exponential **(A)** and power law **(B)** models. The horizontal dashed line indicates 1.000 AU/mL.

Supplementary Table 1 AIC comparison of exponential Vs. power law model.

Supplementary Table 2 Statistics for the estimated number of days in which each group would reach an anti-RBD IgG titer of 1000 AU/mL according to the power law model in terms of mean, standard deviation (SD) and median. Due to the nature of this model, the estimations over extended periods of time display asymptotic behavior.
