# Supplementary material for: Mathematical modelling of the waning of anti-RBD IgG SARS-CoV-2 antibody titers after a two-dose BNT162b2 mRNA vaccination
Source: Front Immunol. 2023 Jan 26;14:1097747. doi: 10.3389/fimmu.2023.1097747 (PMC9909695; doi:10.3389/fimmu.2023.1097747)
Supplement: Supplementary 1 — Time-course of anti-RBD IgG antibody titers for each individual HCW. (A) Individual time-courses of naïve subjects adjusted by exponential model. (B) Individual time-courses of experienced subjects adjusted by exponential model. (C) Individual time-courses of naïve subjects adjusted by power law model. (D) Individual time-courses of experienced subjects adjusted by power law model. [file Presentation_1.pptx]

## Slide 1
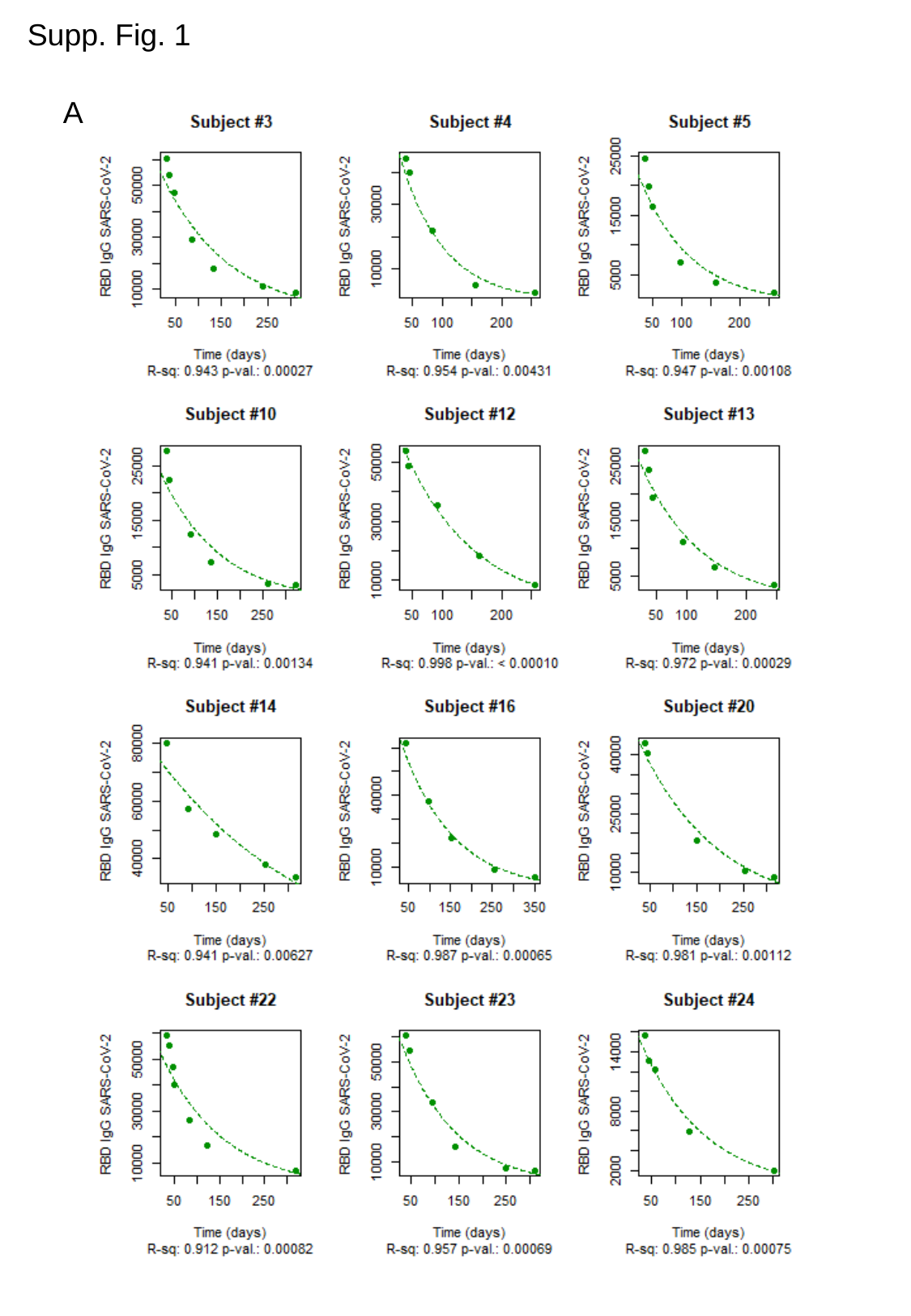

Supp. Fig. 1
A

## Slide 2
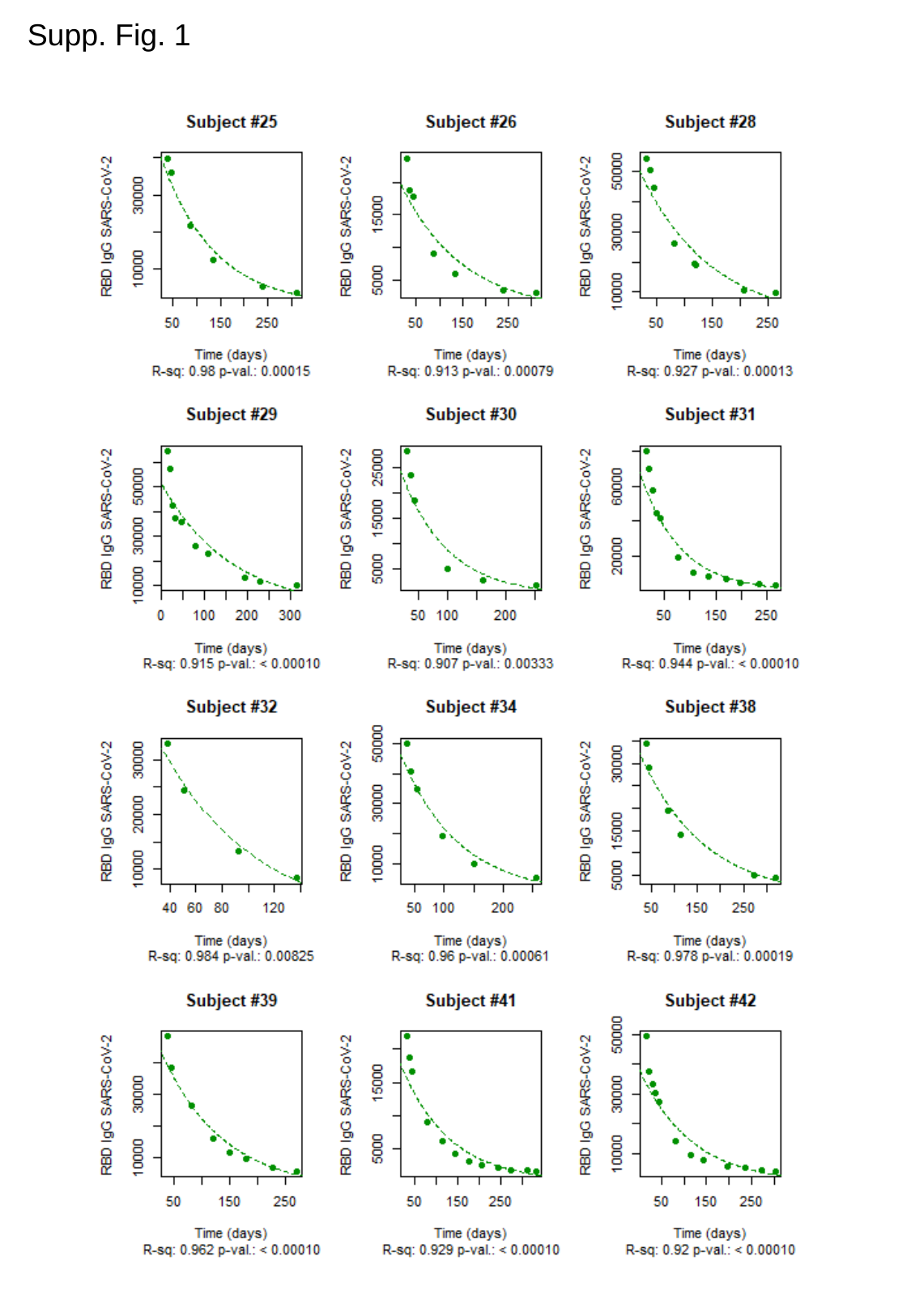

Supp. Fig. 1

## Slide 3
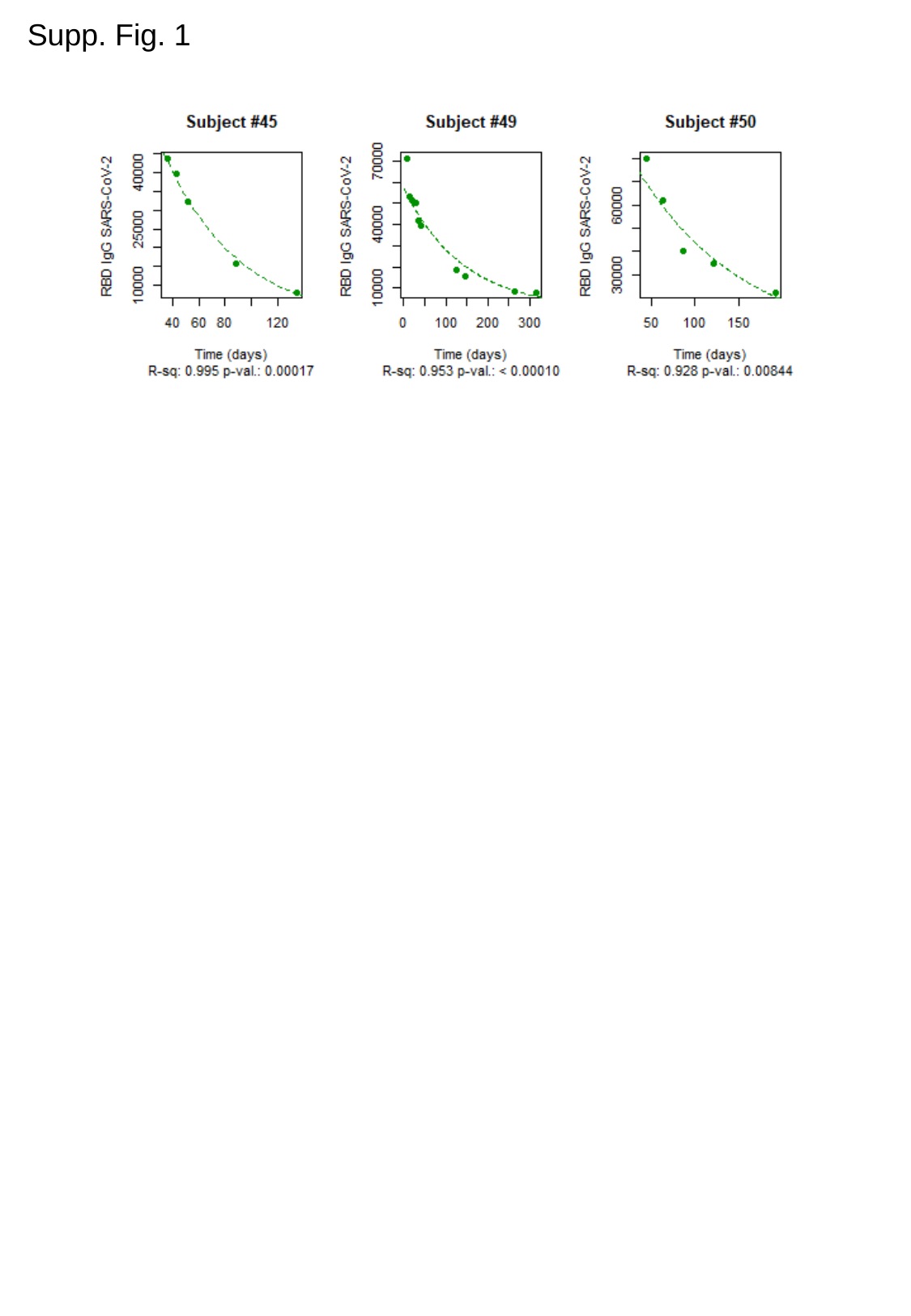

Supp. Fig. 1

## Slide 4
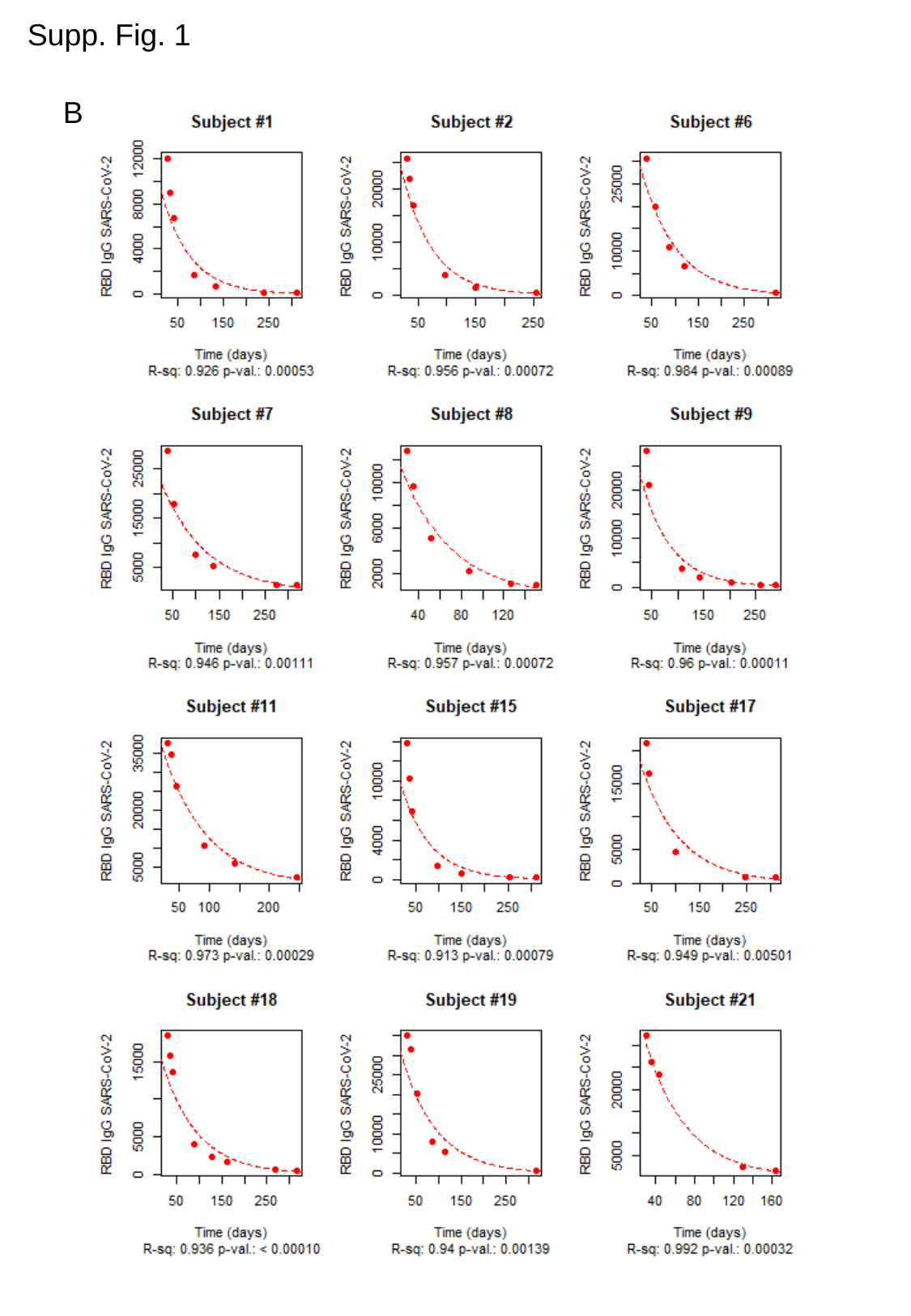

Supp. Fig. 1
B

## Slide 5
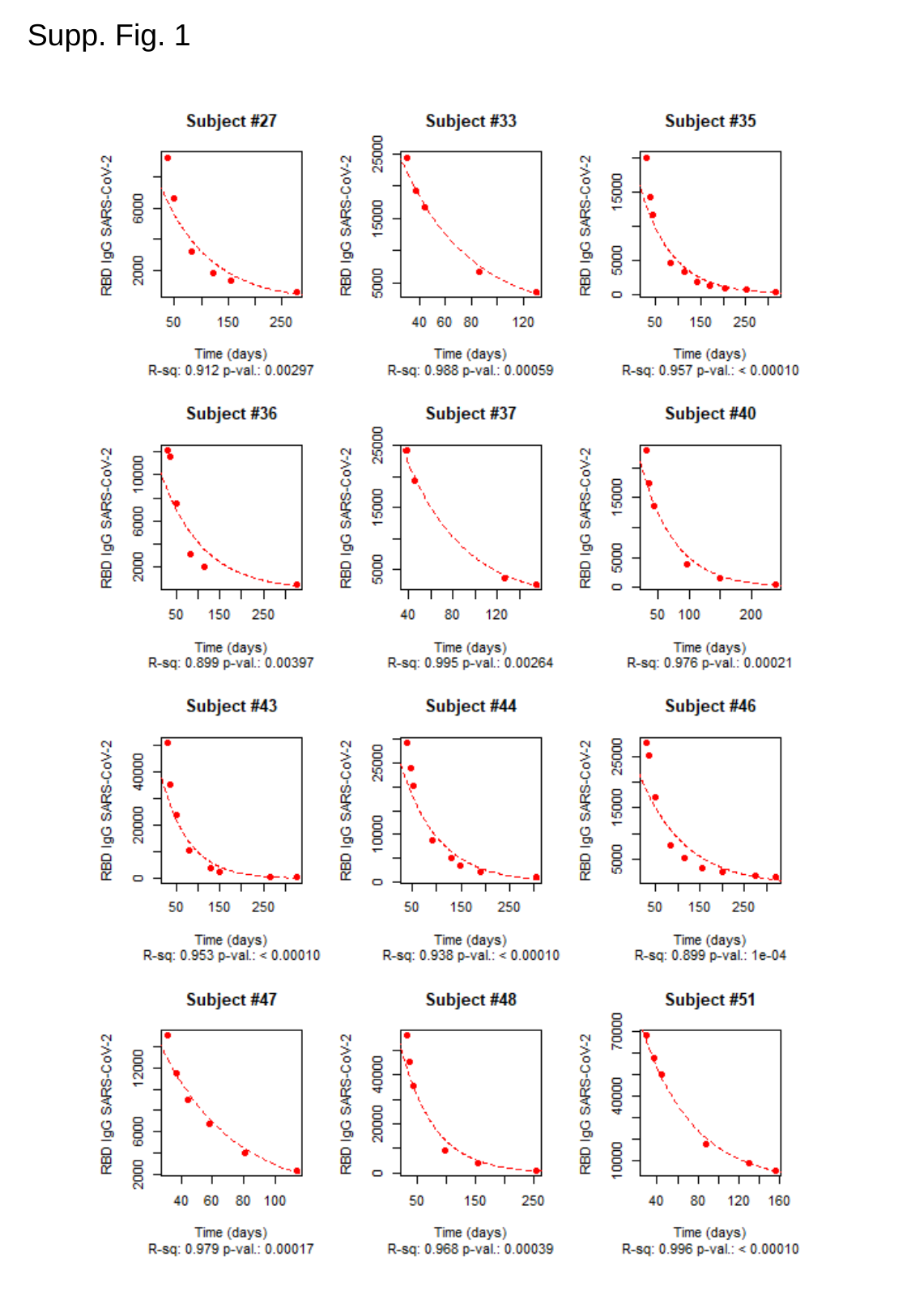

Supp. Fig. 1

## Slide 6
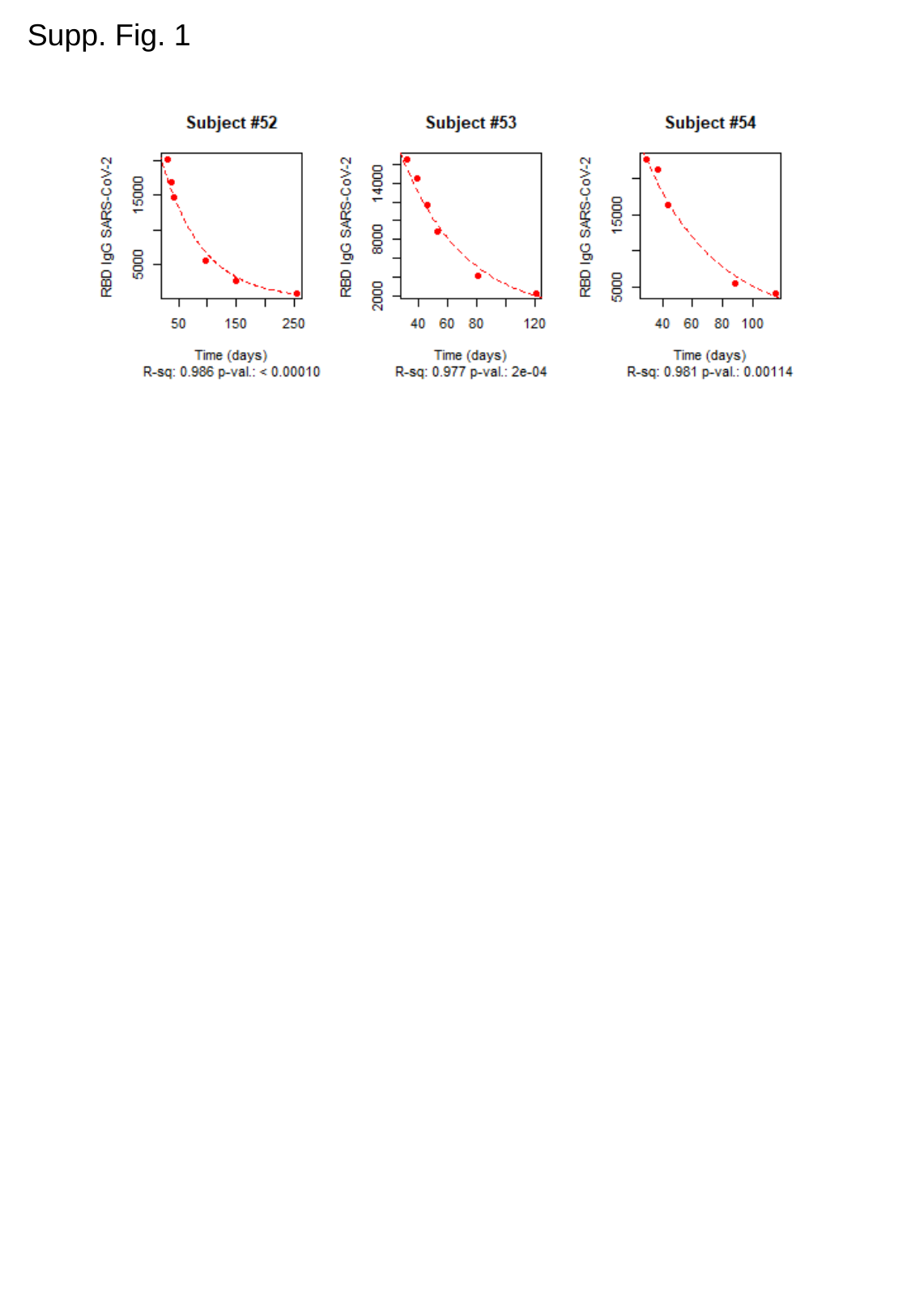

Supp. Fig. 1

## Slide 7
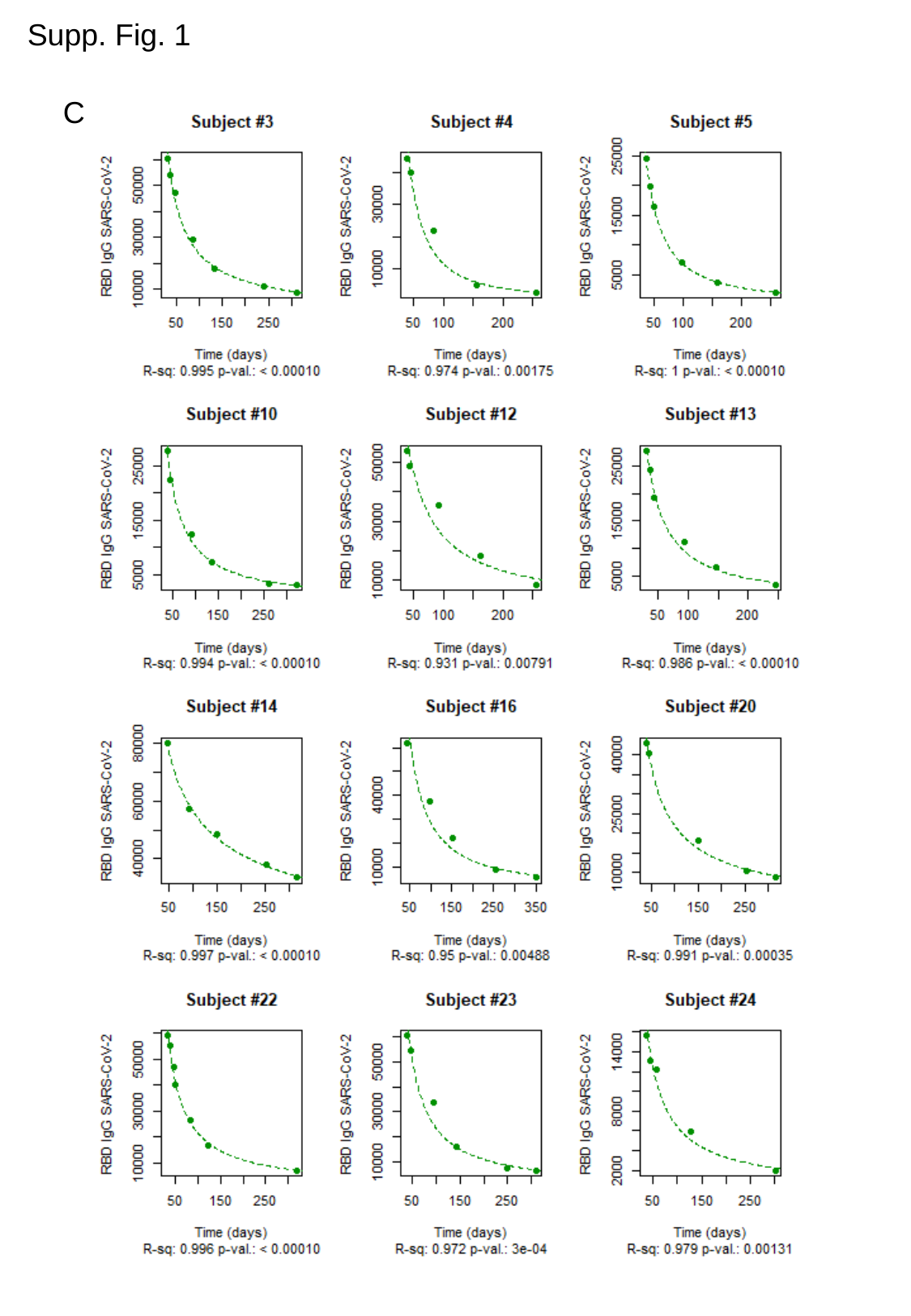

Supp. Fig. 1
C

## Slide 8
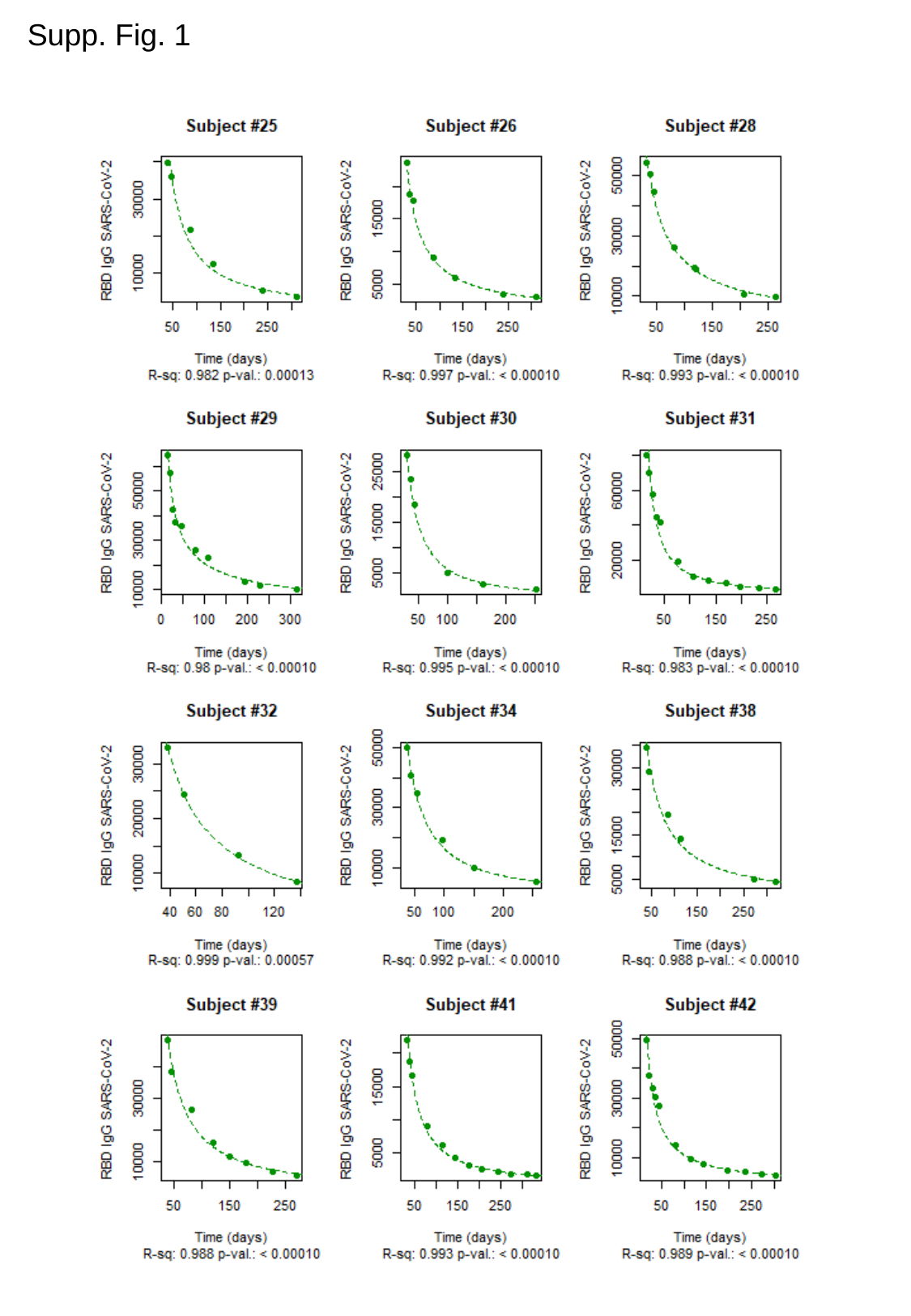

Supp. Fig. 1

## Slide 9
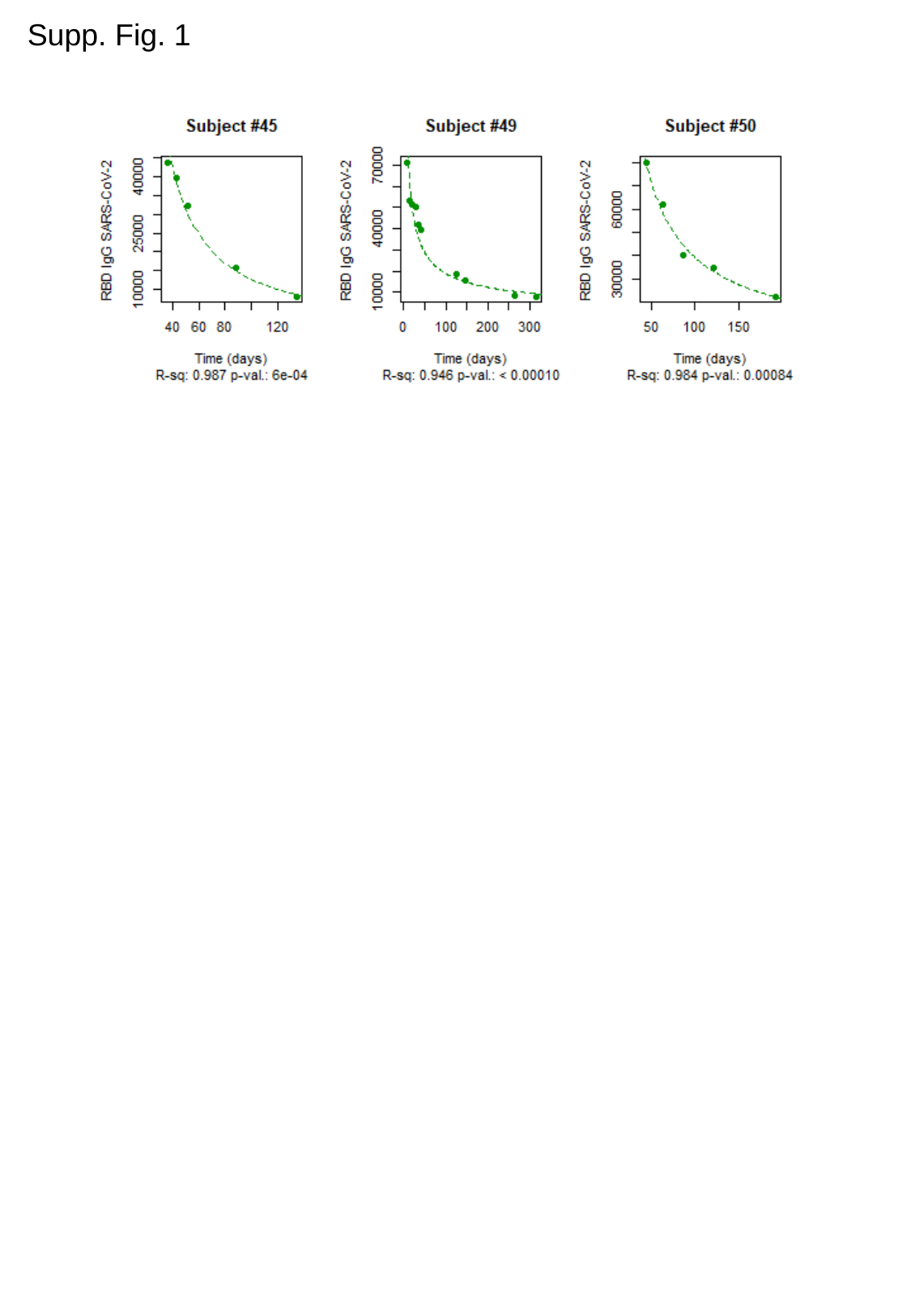

Supp. Fig. 1

## Slide 10
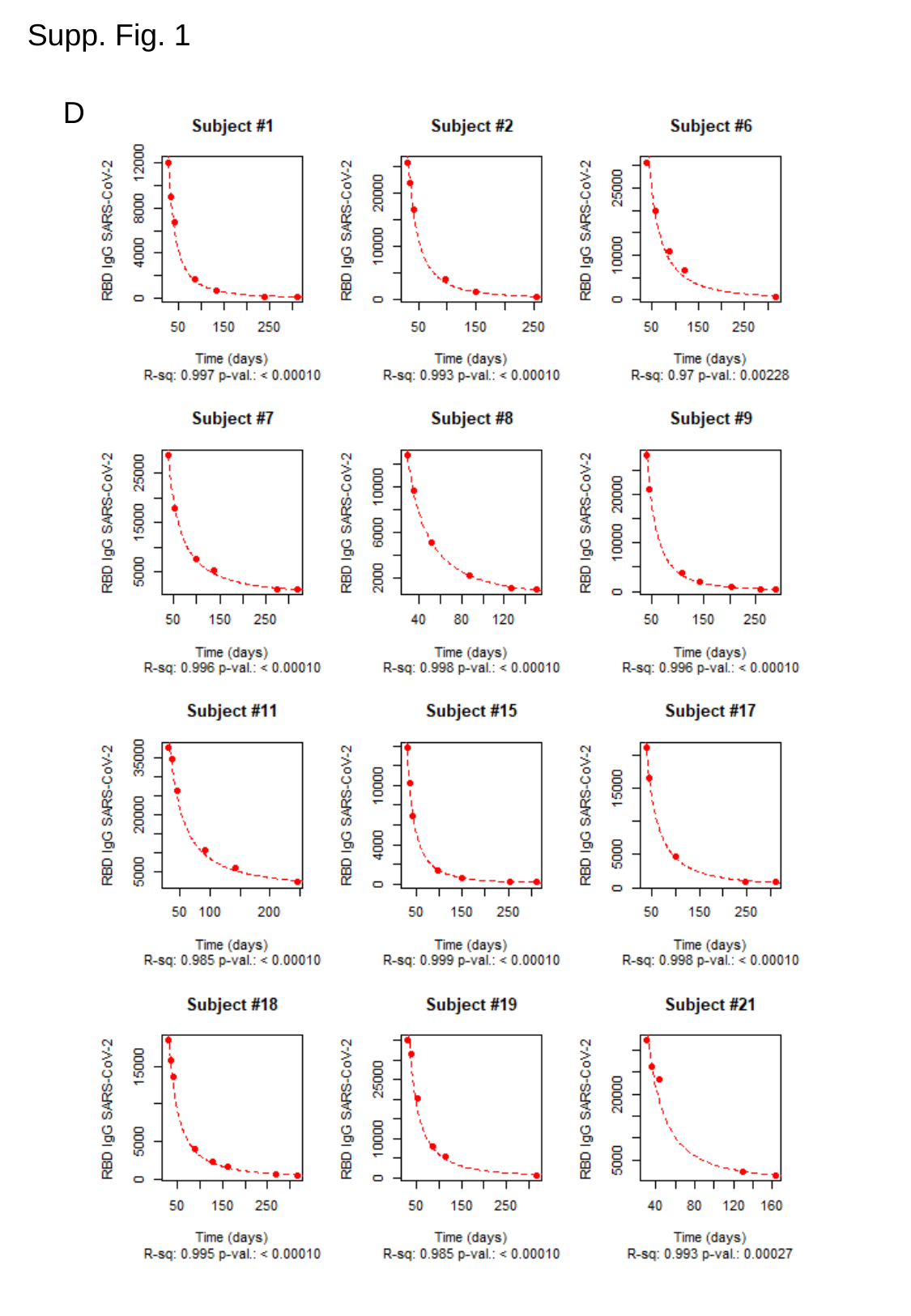

Supp. Fig. 1
D

## Slide 11
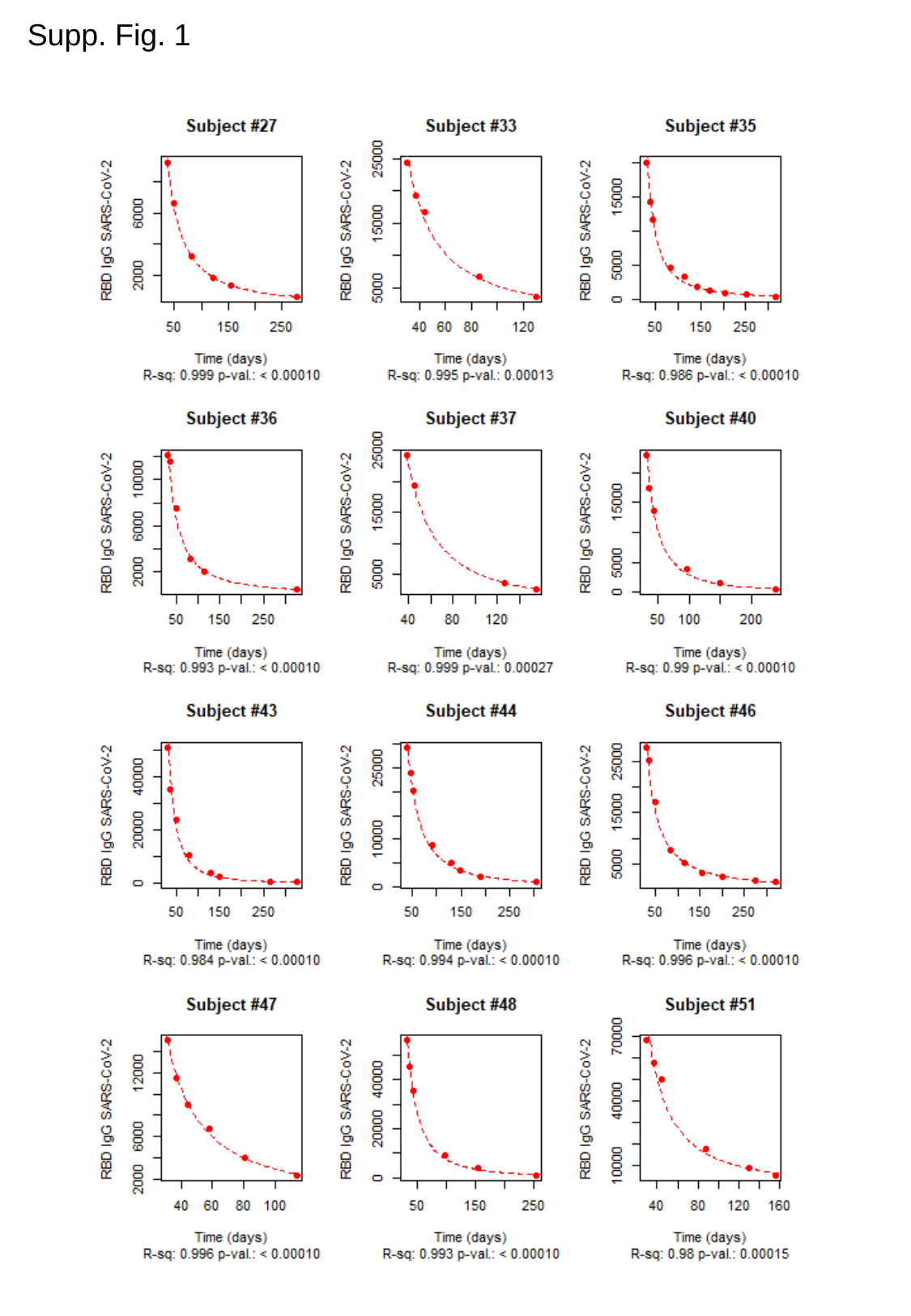

Supp. Fig. 1

## Slide 12
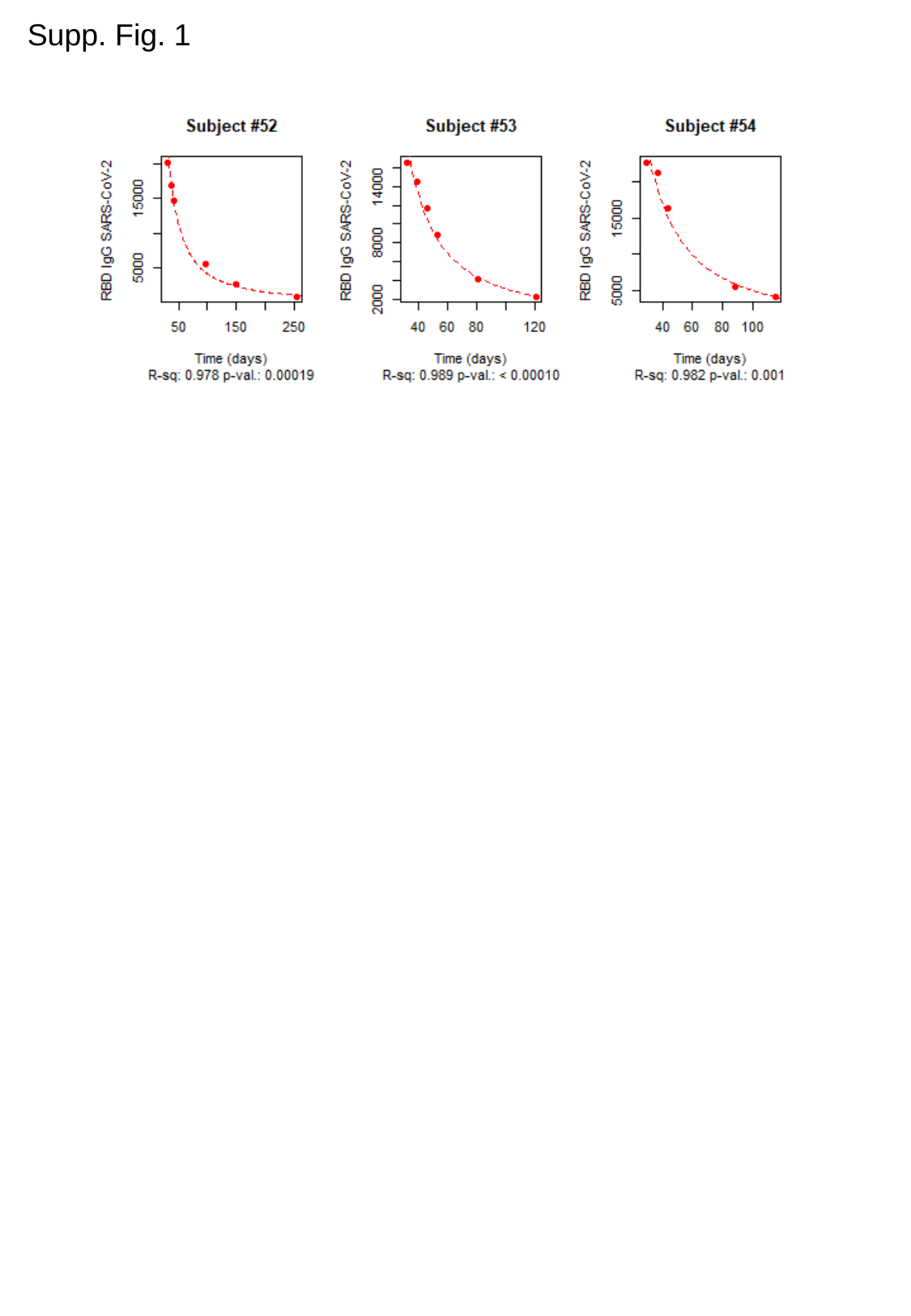

Supp. Fig. 1

## Slide 13
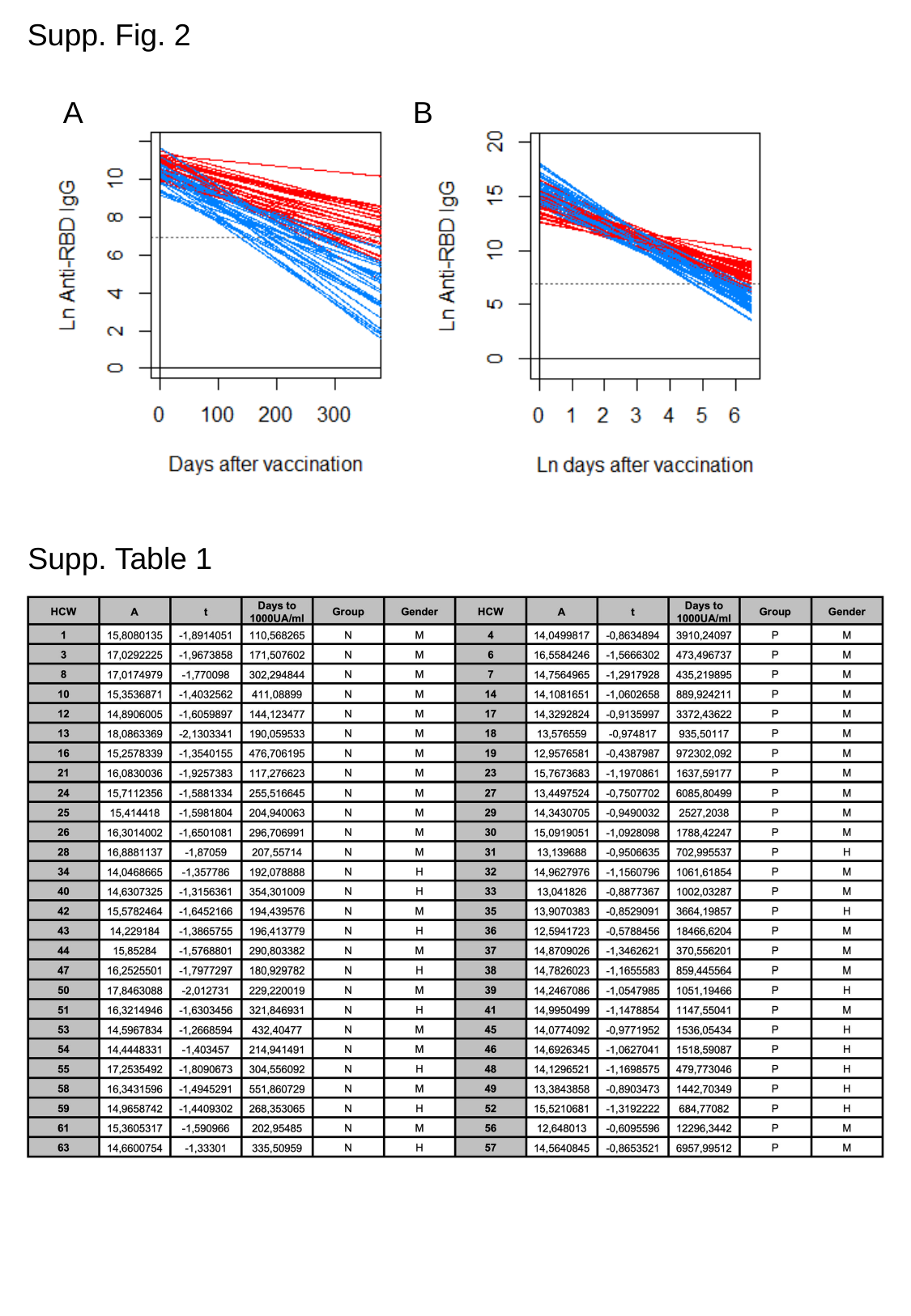

Supp. Fig. 2
A
B
Supp. Table 1

## Slide 14
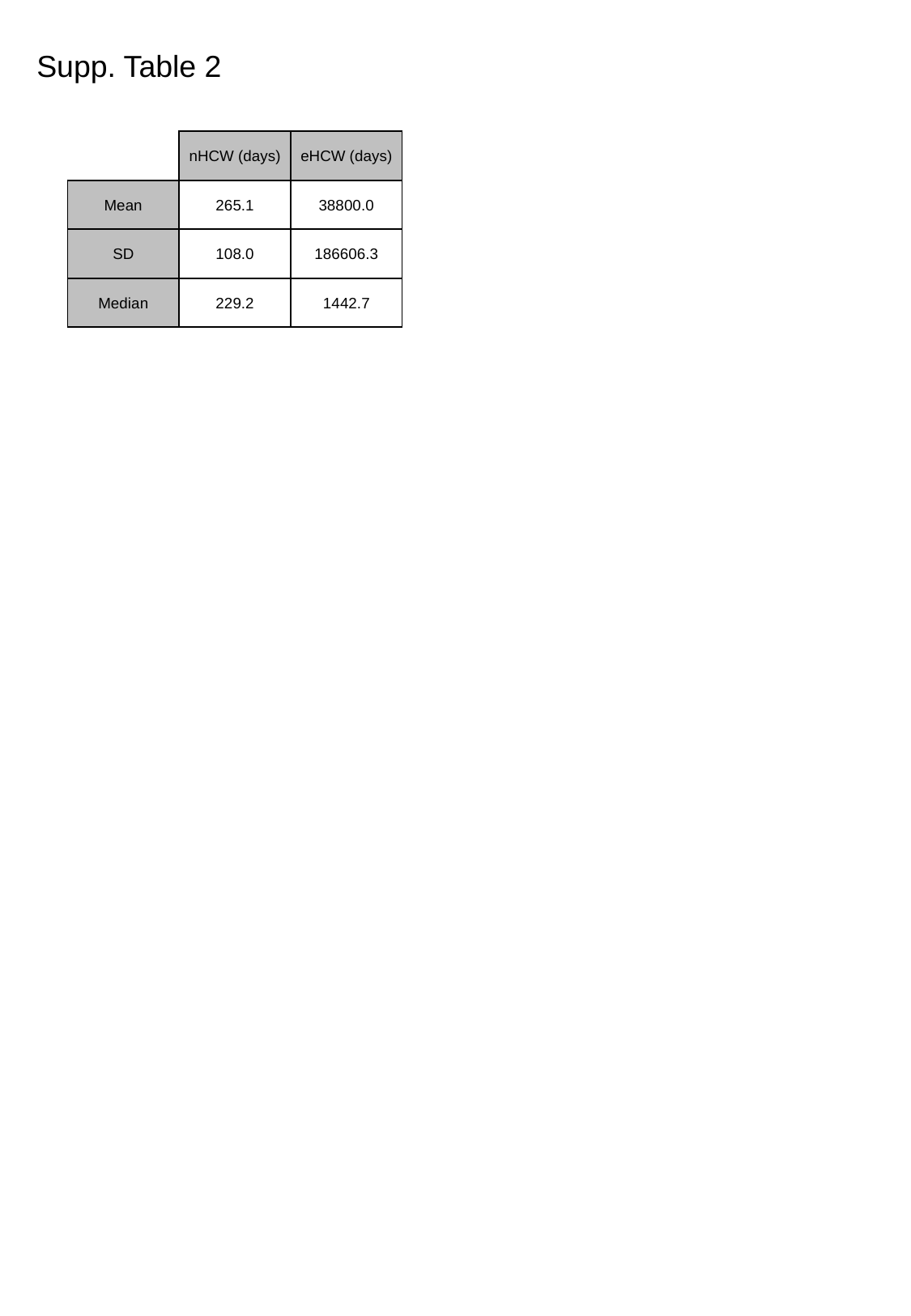

Supp. Table 2
| | nHCW (days) | eHCW (days) |
| --- | --- | --- |
| Mean | 265.1 | 38800.0 |
| SD | 108.0 | 186606.3 |
| Median | 229.2 | 1442.7 |
